# Adaptation of the *Romanomermis culicivorax* CCA-Adding Enzyme to Miniaturized Armless tRNA Substrates

**DOI:** 10.3390/ijms21239047

**Published:** 2020-11-28

**Authors:** Oliver Hennig, Susanne Philipp, Sonja Bonin, Kévin Rollet, Tim Kolberg, Tina Jühling, Heike Betat, Claude Sauter, Mario Mörl

**Affiliations:** 1 Institute for Biochemistry, Leipzig University, Brüderstraße 34, 04103 Leipzig, Germany; oliver.hennig@uni-leipzig.de (O.H.); susanne.philipp@uni-leipzig.de (S.P.); sonja.bonin@uni-leipzig.de (S.B.); k.rollet@ibmc-cnrs.unistra.fr (K.R.); tim.kolberg@uni-leipzig.de (T.K.); tina.md@gmail.com (T.J.); heike.betat@uni-leipzig.de (H.B.); 2 Architecture et Réactivité de l’ARN, Université de Strasbourg, CNRS, IBMC, 67084 Strasbourg, France; c.sauter@ibmc-cnrs.unistra.fr

**Keywords:** CCA-adding enzyme, co-evolution, evolutionary plasticity, minimalized armless tRNAs, tRNA nucleotidyltransferase

## Abstract

The mitochondrial genome of the nematode *Romanomermis culicivorax* encodes for miniaturized hairpin-like tRNA molecules that lack D- as well as T-arms, strongly deviating from the consensus cloverleaf. The single tRNA nucleotidyltransferase of this organism is fully active on armless tRNAs, while the human counterpart is not able to add a complete CCA-end. Transplanting single regions of the *Romanomermis* enzyme into the human counterpart, we identified a beta-turn element of the catalytic core that—when inserted into the human enzyme—confers full CCA-adding activity on armless tRNAs. This region, originally identified to position the 3′-end of the tRNA primer in the catalytic core, dramatically increases the enzyme’s substrate affinity. While conventional tRNA substrates bind to the enzyme by interactions with the T-arm, this is not possible in the case of armless tRNAs, and the strong contribution of the beta-turn compensates for an otherwise too weak interaction required for the addition of a complete CCA-terminus. This compensation demonstrates the remarkable evolutionary plasticity of the catalytic core elements of this enzyme to adapt to unconventional tRNA substrates.

## 1. Introduction

tRNAs are the essential adaptor molecules which enable the decoding of the nucleic acid code into the amino acid sequence during the translational process [1]. To fulfill this function, they need to undergo several maturation steps and interact with the translational machinery [2,3,4,5]. In eukaryotes, this also includes the corresponding enzymes and proteins of mitochondria and chloroplasts [6,7]. For the efficient recognition by a wide range of processing enzymes, translation factors as well as ribosomes, tRNAs fold into a conserved cloverleaf-like secondary structure consisting of acceptor stem, anticodon arm as well as D- and T-arm that adopts an equally conserved three-dimensional L-shape [8,9,10]. The 3′-terminal CCA-triplet of the acceptor stem is a prerequisite for aminoacylation and the correct positioning of the charged tRNA in the ribosome [11,12]. In eukaryotes, this triplet is not genomically encoded but is added post-transcriptionally by tRNA nucleotidyltransferase (CCA-adding enzyme) [13,14,15].

**Figure 1:**
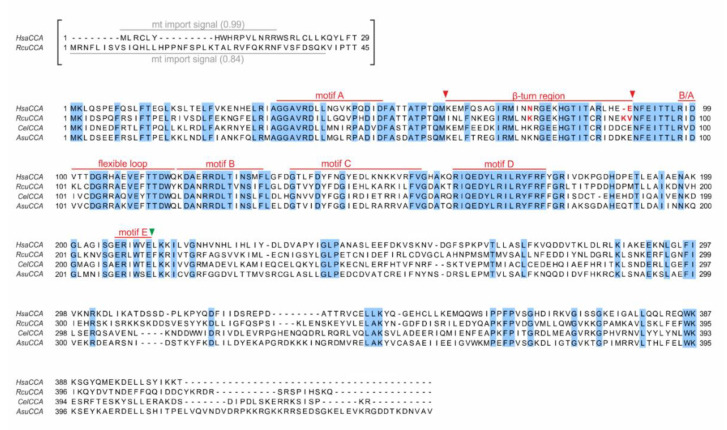
Sequence alignment of CCA-adding enzymes from *Homo sapiens, Romanomermis culicivorax, Caenorhabditis elegans*, and *Ascaris suum*. Light blue positions indicate identical residues. The predicted mitochondrial import signals for *H. sapiens* CCA (*Hsa*CCA) and the *R. culicivorax* CCA-adding enzyme (*Rcu*CCA) (grey bars, import probability is given in brackets) are shown in brackets and were excluded from the cloned open reading frames. Catalytically important elements are labeled in red. Fusion position of reciprocal chimeras A and B are indicated by a green arrowhead. Fusion positions of chimera E (β-turn element) are indicated by red arrowheads (K/I61–E/V90). Mutations K74N and K89Δ/V90E introduced in *Rcu*CCA and N74K and K89ins/E90V in *Hsa*CCA are indicated in red.

tRNA nucleotidyltransferases represent essential enzymes and are ubiquitously found in all domains of life. Representing members of the polymerase β superfamily, they split up into two classes, based on the composition of their catalytic core [16]. Archaeal CCA-adding enzymes represent class I, while their bacterial and eukaryotic counterparts belong to class II [16]. The overall sequence identity among both tRNA nucleotidyltransferase classes is rather low, although they catalyze the same reaction [17]. The catalytic core motif common in both classes consists of two aspartate residues DxD (x, any amino acid) that coordinate the catalytically important metal ions [18,19,20]. In class II enzymes, the DxD sequence belongs to motif A, one of the five conserved motifs A to E located in the N-terminal part of this tRNA nucleotidyltransferase type (Figure 1) [21]. Motif A binds two Mg^2+^ ions required for nucleotide transfer onto the tRNA substrate via the general two metal ion mechanism of polymerases [19]. Motif B is involved in ribose binding [21], while motif C is a flexible element which coordinates interdomain movements, contributing to the proper orientation of the substrates within the active center [22,23]. Motif D represents an amino acid-based template, where arginine and aspartate residues form Watson–Crick-like hydrogen bonds with the incoming nucleotides, and their orientation in the catalytic core determines the specificity for CTP or ATP, respectively [21]. Lastly, motif E stabilizes a helix-turn structure in motif D and is discussed to interact with the tRNA primer strand in the catalytic core [21,24].

Another catalytically important region is a flexible loop consisting of 10–20 amino acids that is located immediately upstream of motif B. While it is not conserved at the sequence level [25], its interaction with the amino acid template of motif D is required for the specificity switch from C to A incorporation, where it acts as a lever to accommodate the ATP in the nucleotide binding pocket [22,26]. Between motif A and the flexible loop, a β-turn element was identified that is also involved in A-addition, as it binds and positions the priming 3′-end of the growing CCA terminus in the catalytic core [22]. Similar β-turn regions are present in many different polymerases, ranging from both classes of tRNA nucleotidyltransferases to poly(A) polymerases and DNA polymerases, underscoring the catalytically important function of this element [27,28,29,30]. For a structural view of the reaction mechanism, we refer to Tomita and Yamashita, 2014 [31].

All these conserved motifs build up the active site in the N-terminal part of the enzyme. The C-terminus, in contrast, is much less conserved. Yet, it is of functional importance, as it is involved in tRNA binding, where it anchors the TΨC loop of the L-shaped tRNA substrate during nucleotide addition [24,32,33,34,35]. Correspondingly, artificial CCA-adding substrates like mini- or microhelices are recognized and accepted for CCA incorporation at a much lower efficiency [36,37]. Yet, metazoan mitochondria carry tRNA molecules that deviate from the cloverleaf structure, lacking either the D- or the T-arm [38,39,40]. As an example, the mammalian mt-tRNA^Ser^(AGY) lacks the complete D-arm, so that D- and T-arm interactions do not exist [41,42]. In the mitochondrial genomes of nematodes, acari and arachnids, this situation comes to an extreme. These genomes are rich in genes for tRNAs that lack either the D- or the T-arm or even both [38,43,44,45,46]. In the mermithid *Romanomermis culicivorax*, mt-tRNA molecules with the most dramatic truncations were identified, resulting in miniaturized hairpin-like tRNAs with a length of down to 45 nts, in contrast to the standard average tRNA size of 76 nts [47,48]. Such extremely truncated tRNAs fold into a three-dimensional boomerang-like shape that deviates from the consensus L-form [47]. Yet, these organisms encode for a single CCA-adding enzyme that has to act on both cytosolic as well as mitochondrial tRNA pools [49,50,51,52,53], and it was shown for the corresponding enzyme of *Caenorhabditis elegans* that it recognizes mt-tRNAs lacking D- or T-arm [52]. Since in *R. culicivorax* nine mt-tRNAs lack both arms, representing the strongest deviation from the consensus structure [45], we investigated the co-evolution and substrate adaptation of its CCA-adding enzyme. In a comparative analysis, we identified the β-turn element as a major adaptation to the hairpin-like tRNA substrates. This adaptation is based on an increased substrate affinity of the enzyme. Hence, while the conventional substrate binding based on interactions between the enzyme’s C-terminus and the TΨC loop/T-arm is not possible with such tRNA hairpins, a different part of the enzyme took over this function to assure a sufficiently strong substrate binding for CCA-addition, demonstrating a surprising evolutionary plasticity of these enzymes.

## 2. Results

### 2.1. RcuCCA Adds a Complete CCA-Triplet to Armless and Canonical tRNAs

To investigate the catalytic activity and substrate specificity of the *R. culicivorax* CCA-adding enzyme (*Rcu*CCA), we identified a corresponding singular open reading frame in the *R. culicivorax* genome assembly available at https://parasite.wormbase.org/Romanomermis_culicivorax_prjeb1358/Info/Index/ [54]. The sequence conservation was investigated in an alignment with the corresponding human enzyme (*Hsa*CCA). As it was reported that the CCA-adding enzyme of *Caenorhabditis elegans* is adapted to the bizarre mitochondrial tRNAs of this organism [52], the sequence of this enzyme as well as that of *Ascaris suum* (another nematode with mt-tRNAs lacking D- or T-arm [38,39,43]) were included (Figure 1). At the amino acid level, the overall sequence identity and similarity between *Rcu*CCA and *Hsa*CCA is 48% and 66%, respectively. Carrying the complete active site as well as a putative mitochondrial import sequence as predicted [55], the N-terminus shows a comparatively higher conservation (78% sequence similarity), as it is true for the corresponding enzymes of *C. elegans* and *A. suum* (Figure 1). In the less conserved C-terminal part, *Rcu*CCA and the other nematode enzymes carry a short insertion and a terminal extension.

**Figure 2:**
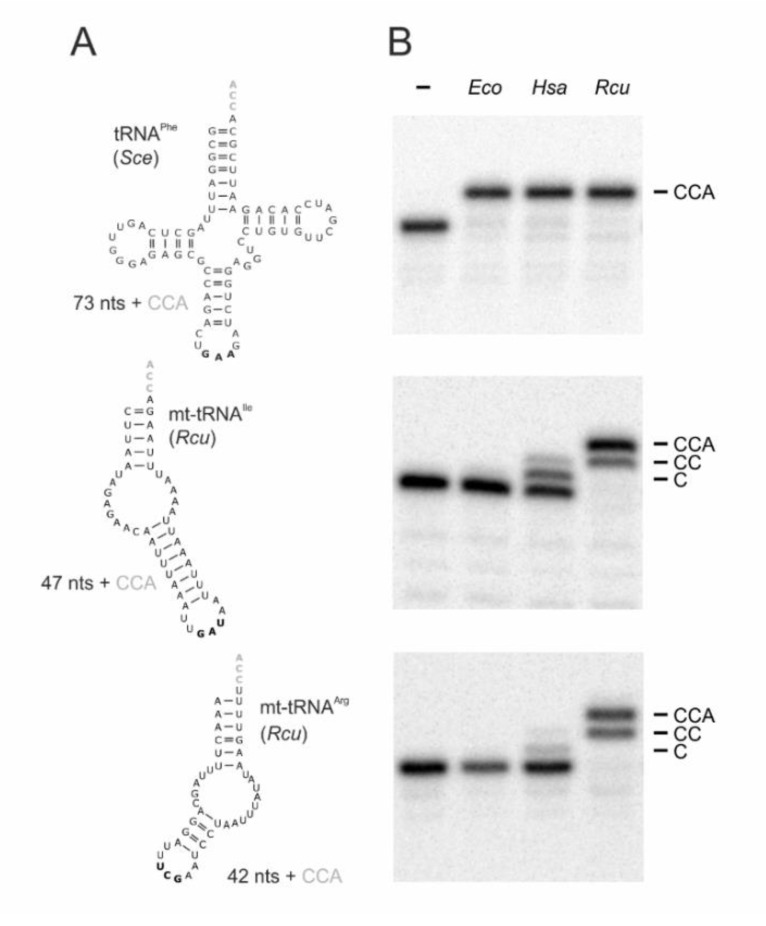
CCA-addition on conventional and hairpin-like tRNA substrates. (**A**) tRNA^Phe^ from *Saccharomyces cerevisiae* (*Sce*) represents a conventionally structured tRNA substrate of standard size (73 nts without CCA), while the mitochondrial tRNAs for isoleucine and arginine from *R. culicivorax* (*Rcu*) considerably deviate in size (47 and 42 nts, respectively; both without 3′-terminal CCA-triplet) and structure, lacking both D- and T-arms. Anticodons are indicated in bold. (**B**) CCA-addition on radioactively labeled tRNA transcripts catalyzed by the corresponding enzymes (20 ng each) of *Escherichia coli* (*Eco*), *H. sapiens* (*Hsa*), and *R. culicivorax* (*Rcu*). Incubation without enzymes represent negative controls (−). All enzymes completely convert the canonical tRNA^Phe^ from *S. cerevisiae* into a mature transcript with CCA-end. On armless mt-tRNAs, the *E. coli* enzyme shows no activity at all, while the human enzyme adds only one or two C residues to mt-tRNA^Arg^ and mt-tRNA^Ile^, respectively. In contrast, the enzyme of *R. culicivorax* readily synthesizes a complete CCA-end on both transcripts, although the time of incubation was not sufficient for 100% A-addition. The experiment was done in three independent replicates. The panel shows a representative autoradiogram.

A conserved methionine residue downstream of the mt import signal was chosen as the N-terminus of the expressed open reading frame (labeled as position 1 in Figure 1). In previous experiments on the human enzyme, this position was successfully used and the absence of the mt import signal had no effect on its catalytic activity [37,50,56,57]. The open reading frame was synthesized as a codon-optimized DNA sequence and recombinantly expressed in *Escherichia coli*. Together with the corresponding enzymes from *E. coli* (*Eco*CCA; an organism exclusively carrying conventional cloverleaf-like tRNAs) and *Homo sapiens* (*Hsa*CCA; an organism carrying conventional cytosolic as well as moderately reduced mt-tRNAs), the purified enzyme was tested in vitro for activity. As substrates, three different radioactively labeled tRNA transcripts were generated by in vitro transcription (Figure 2A), as it is well established that tRNA nucleotidyltransferases from all kingdoms readily accept in vitro transcripts lacking base modifications [25,58,59,60]. tRNA^Phe^ from *Saccharomyces cerevisiae* is one of the best characterized tRNAs and represents a standard substrate for many tRNA-interacting enzymes [58,61,62], since the unmodified in vitro transcript folds into a structure almost identical to the native tRNA [63,64]. Furthermore, two armless mt-tRNAs from *R. culicivorax* were generated. With a length of 42 nts and 47 nts, respectively, mt-tRNA^Arg^ and mt-tRNA^Ile^ represent the shortest tRNAs identified so far, and the in vitro transcripts fold into hairpin-like structures with two single-stranded connector elements replacing D- and T-arm (Figure 2A) [47]. On the standard tRNA^Phe^, all enzymes added a complete CCA-triplet, indicating highly active enzyme preparations (Figure 2B). On the armless mt-tRNA substrates, however, the bacterial enzyme *Eco*CCA was completely inactive and did not add any nucleotides. The human enzyme *Hsa*CCA that has to recognize the human D-arm-lacking mt-tRNA^Ser^(AGY) (Figure S1) catalyzed a moderate incorporation of two residues on the armless *Rcu* mt-tRNA^Ile^, but was almost inactive on the even smaller *Rcu* mt-tRNA^Arg^. In contrast, *Rcu*CCA added complete CCA-triplets, regardless whether the substrate represented a conventional cloverleaf-structured tRNA or an armless hairpin-like tRNA, indicating an efficient adaptation to these miniaturized substrates (Figure 2B).

To investigate the substrate preferences of *Hsa*CCA and *Rcu*CCA in more detail, a Michaelis–Menten kinetics analysis was performed. Due to the limited RNA solubility, excessive saturating amounts of tRNA cannot be used, and the obtained parameters represent apparent values [24,25,30,58,65]. As *Eco*CCA showed no activity on armless tRNAs, this enzyme was excluded from further analysis. To discriminate between C- and A-addition, assays were performed on tRNAs lacking the CCA-end in the presence of either α-^32^P-CTP or α-^32^P-ATP and unlabeled NTPs [23,37,66]. As shown in Table 1, the kinetic parameters of both enzymes on tRNA^Phe^ are rather similar for CC-incorporation, and *Rcu*CCA is somewhat less efficient (0.5-fold) in adding the complete CCA terminus (CCA*). On the armless mt-tRNAs for isoleucine and arginine, *Hsa*CCA and *Rcu*CCA are almost equally active in CC-addition, with somewhat higher values for *Rcu*CCA (1.2 to 1.7x). However, when the complete CCA-incorporation is investigated (CCA*), the *Romanomermis* enzyme is much more efficient on mt-tRNA^Ile^ and mt-tRNA^Arg^ (3.3 to 10x). As the obtained k_cat_ values for *Hsa*CCA in this reaction are at the detection limit, the actual efficiency of *Rcu*CCA relative to *Hsa*CCA is probably much higher.

Taken together, both enzymes prefer a conventionally structured tRNA as substrate. On the hairpin-like tRNAs, *Rcu*CCA still adds complete CCA-ends, although at a lower efficiency for mt-tRNA^Arg^. The human enzyme, however, strongly prefers the conventional tRNA and is severely affected in A-addition on armless tRNAs, resulting in incomplete and hence non-functional tRNA molecules as already seen in CCA-addition assay (Figure 2B).

### 2.2. In the Romanomermis Enzyme, Especially the Catalytic Core is Adapted to Armless tRNA Substrates

**Figure 3:**
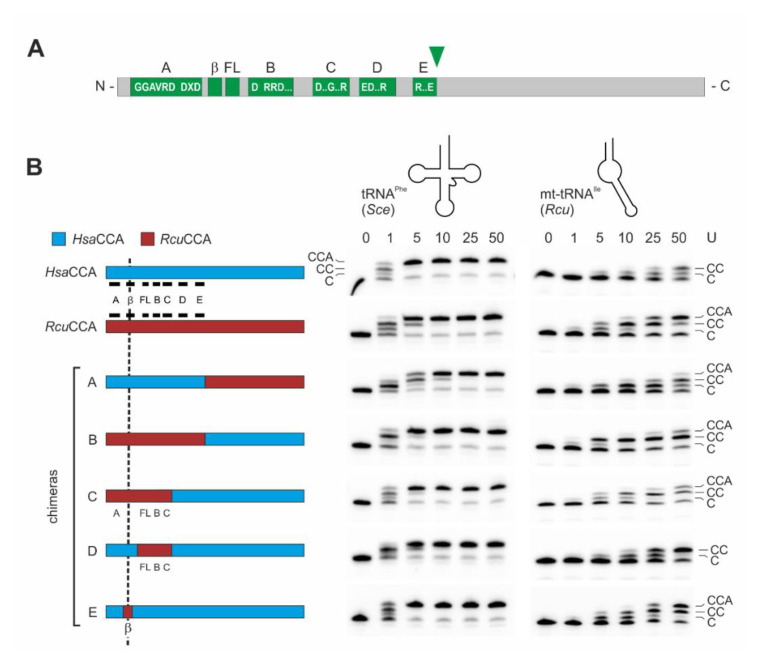
Catalytic activity of wild-type and chimeric enzymes on tRNA^Phe^ and mt-tRNA^Ile^. (**A**) Bar representation of class II CCA-adding enzymes. The N-terminal region contains the catalytic core consisting of five motifs A to E, a β-turn element (β) and a flexible loop (FL). The green arrowhead indicates the fusion position for enzyme chimeras A and B. (**B**) Left: Bar representation of tested enzyme chimeras consisting of *Hsa*CCA (cyan) and *Rcu*CCA (red) regions. Catalytic core elements are indicated in black. The reciprocal chimeras A and B are fused after position E213, downstream of motif E. The replaced β-turn element (β) in chimera E is located between motif A and the flexible loop (FL). **Gel panels:** CCA-addition on the conventional tRNA^Phe^ from yeast (*Sce*, left) and the armless mt-tRNA^Ile^ from *R. culicivorax* (*Rcu*, right) with increasing amounts of enzymes indicated as arbitrary units (U). All enzymes catalyze an efficient CCA-incorporation on the conventional tRNA. On the armless tRNA^Ile^, the *Romanomermis* wt enzyme synthesizes a complete CCA-end, while the corresponding human enzyme adds only two C-residues. Chimeras A and B also add the terminal A, but at a somewhat reduced level. On this tRNA substrate, chimera B, carrying the catalytic core of *Rcu*CCA, is more efficient than chimera A, where A-incorporation is only visible at the highest enzyme concentration. Chimera C still shows full CCA-addition, whereas no terminal A-incorporation is observed for chimera D. Chimera E shows an efficiency comparable to that of the *Rcu*CCA wt enzyme, indicating the importance of the β-turn region in the reaction on armless tRNAs. The fact that chimera E is more active than chimera B likely reflects differences in the compatibility of the chosen fusion positions in these chimeras, resulting in different protein folding and/or catalytic efficiency. For each construct, up to four independent experiments were performed. For each tRNA substrate, a representative gel is shown.

To identify the contribution of individual enzyme regions to the recognition of armless tRNAs as substrates for CCA-addition, we followed a strategy that we successfully applied to investigate several CCA-adding enzymes concerning their enzymatic features [33,58,67]. We reciprocally exchanged N- and C-termini of *Hsa*CCA and *Rcu*CCA, carrying the complete catalytic core and the region involved in tRNA binding, respectively. Based on the sequence alignment shown in Figure 1, we selected a glutamate at position 212 (*Hsa*CCA) and 213 (*Rcu*CCA), representing the last invariant residue of motif E [68] as fusion position (Figure 3A). To allow for a direct comparison of enzymatic activities of the resulting proteins, we adjusted the efficiency of CCA-addition on the canonically structured tRNA^Phe^ substrate and defined an arbitrary unit as the amount of enzyme required for 50% substrate turnover, ranging between 0.3 ng for wild type (wt) enzymes and 0.5 to 1.3 ng for chimeras. Incubation of the standard substrate tRNA^Phe^ with 1 to 50 arbitrary units of both wt enzymes as well as chimera A (N-terminal catalytic core of *Rcu*CCA, C-terminus of *Hsa*CCA) and the reciprocal chimera B indicate that all enzymes are fully active and efficiently synthesize a complete CCA-terminus (Figure 3B, left gel panel). On the minimalized mt-tRNA^Ile^ (Figure 3B, right gel panel), even 50 units of the human wt enzyme added only two C residues. In contrast, the same amount of *Romanomermis* enzyme added a complete CCA-end. Surprisingly, chimera B (with the catalytic core of *Rcu*CCA) synthesized a complete CCA-end at considerable efficiency, while identical units of chimera A, carrying the tRNA-binding C-terminus of *Rcu*CCA, catalyzed the full CCA-addition to a lesser extent.

While these reactions indicate that both enzyme parts participate in the adaptation to the armless tRNA substrates, the contribution of the catalytic core seems to have a greater impact on the acceptance of these substrates. Based on these results, we generated a series of chimeric enzymes carrying parts of the *Rcu*CCA catalytic core in the context of the human enzyme (Figure 3B, Table S1). For chimera C, the human C-terminal part was extended to include motifs D and E. Comparable to chimera B, this construct added the full CCA-end to *Rcu* mt-tRNA^Ile^. As the flexible loop (FL) and motif C are involved in the enzymes’ specificity switch to A-addition [22,23,26], chimera D was constructed, consisting of *Hsa*CCA where the region spanning these two elements was replaced by the corresponding part from *Rcu*CCA. While this enzyme was fully active on tRNA^Phe^, the loop and motif C replacement did not result in A-addition on mt-tRNA^Ile^. These results led to the conclusion that an element upstream of the flexible loop must be responsible for A-addition on armless tRNA substrates. As this region contains a small β-turn element that was described to contribute to tRNA-CC primer positioning and, as a consequence, A-addition [22], chimera E was constructed, where a stretch of 30 amino acid residues (positions 61–91) carrying this element was replaced in the backbone of the human enzyme. The resulting enzyme catalyzed CCA-addition on both tRNA^Phe^ and mt-tRNA^Ile^ at efficiencies comparable to the wt *Rcu*CCA enzyme (Figure 3B). Hence, this divide-and-conquer approach allowed us to identify the β-turn as an important element involved in the adaptation of *Rcu*CCA to the armless tRNA substrates.

### 2.3. The β-Turn of the R. culicivorax CCA-Adding Enzyme Strongly Contributes to Substrate Binding and CCA Incorporation on Armless tRNAs

**Figure 4:**
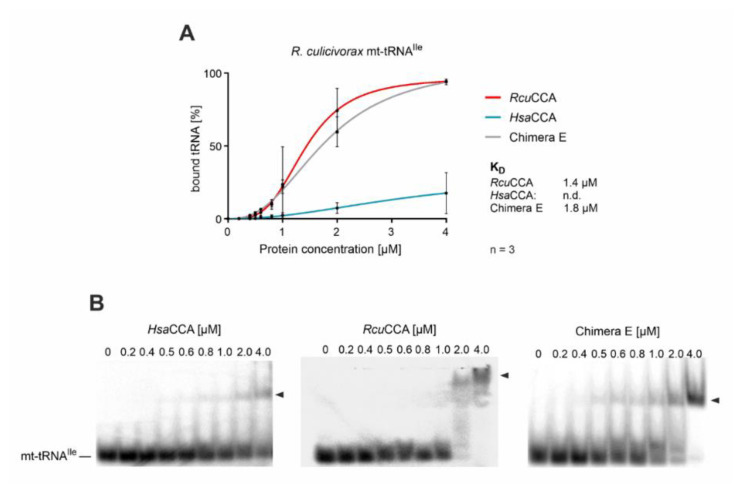
Binding of wt and chimeric CCA-adding enzymes to an armless tRNA. Quantitative analysis of enzyme binding to the armless mt-tRNA^Ile^ determined by electrophoretic mobility shifts. (**A**) While the tRNA interaction of *Hsa*CCA over the whole concentration range (0–4 µM) is too weak to calculate dissociation constants, *Rcu*CCA as well as chimera E exhibit a strong affinity to this substrate, resulting in dissociation constants of 1.4 and 1.8 µM, respectively. Data are means ± SD; *n* = 3. (**B**) Images of representative gel shift assays on *Hsa*CCA, *Rcu*CCA, and chimera E with mt-tRNA^Ile^ as substrate.

To investigate whether the β-turn of *Rcu*CCA contributes to an increased efficiency in binding of armless tRNA substrates, we performed electrophoretic mobility shift experiments on chimera E and the parental enzymes *Hsa*CCA and *Rcu*CCA with an armless tRNA transcript. Radioactively labeled mt-tRNA^Ile^ lacking the CCA terminus was incubated with increasing amounts of recombinantly expressed enzymes and separated by native polyacrylamide gel electrophoresis (Figure 4). Enzyme-bound and free substrates were visualized and binding parameters were determined by nonlinear regression. The *Romanomermis* enzyme showed an efficient and robust binding to mt-tRNA^Ile^, resulting in a K_d_ value of 1.4 µM, while for the human enzyme, no significant binding could be detected, as previously reported for this enzyme class [23,33,69]. Interestingly, and concurrent with its wt-like activity on mt-tRNA^Ile^ (Figure 3B), chimera E also showed a high affinity for mt-tRNA^Ile^ at a K_d_ of 1.8 µM.

When transcripts of mt-tRNA^Ile^ with different 3′-ends (mt-tRNA^Ile^, mt-tRNA^Ile^-C, mt-tRNA^Ile^-CC, mt-tRNA^Ile^-CCA) were offered to *Rcu*CCA, the enzyme bound all substrates with similar affinity, indicating that the composition of the tRNA 3′-end does not affect this interaction (Figure S2A). The human enzyme, in contrast, did not show efficient binding to any of these substrates. Gel shift experiments on the conventional substrate tRNA^Phe^ indicate that this high affinity of the *Rcu*CCA enzyme is not restricted to armless tRNA substrates (Figure S2B). With a binding constant of 1.3 µM, the enzyme exhibits a similar binding behavior to the cloverleaf-shaped tRNA as it does on the armless mt-tRNA^Ile^ (K_d_ = 1.4 µM), while the human enzyme again shows almost no interaction. Hence, the adaptation of the *Romanomermis culicivorax* CCA-adding enzyme to process both cloverleaf-structured cytosolic as well as armless mitochondrial tRNAs is obviously achieved by a general exceptional tight interaction with its substrates, regardless whether they represent canonical or minimalized tRNAs.

**Figure 5:**
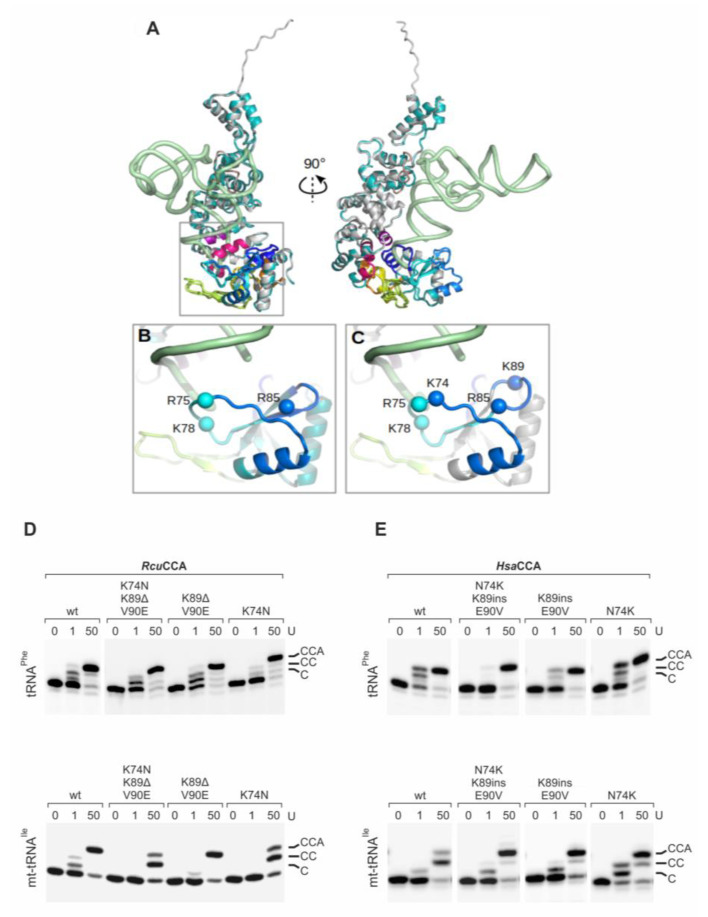
The β-turn in *Hsa*CCA and *Rcu*CCA enzymes. (**A**) Superimposed full-length models of *Hsa*CCA (cyan) and *Rcu*CCA (light gray) with the backbone of a bound tRNA (green) in two perpendicular views. The tRNA position was obtained by superimposing the A-adding enzyme:tRNA-CC complex from *Aquifex aeolicus* onto the human enzyme [34,35,70]. Motif A (dark blue), β-turn region (medium blue), β-turn (light blue), B/A motif and flexible loop (green), motif B (yellow), motif C (orange), motif D (red) and motif E (violet) are indicated. (**B**,**C**). Zoom into the β-turn region and the tRNA 3′-end (corresponding to the squared region in (**A**) of *Hsa*CCA (**B**) and RcuCCA (**C**). Spheres represent the Cα positions of positively charged residues (K and R). *Rcu*CCA carries two additional lysines at positions 74 and 89 that might contribute to tRNA binding and primer positioning. Model is based on the crystal structure of the A-adding enzyme from *Aquifex aeolicus* [34]. (**D**) Enzymatic activity of *Rcu*CCA carrying mutations K74N, K89Δ/V90E and K74N/K89Δ/V90E. 0, 1, and 50 arbitrary units of enzyme variants were incubated with yeast tRNA^Phe^ and the armless mt-tRNA^Ile^. *Rcu*CCA wt accepts both tRNAs for CCA-addition, while *Rcu*CCA K74N is less active on mt-tRNA^Ile^, resulting in a considerably reduced A-addition. In contrast, *Rcu*CCA K89Δ/V90E catalyzes full CCA-addition on the conventional (comparable to wt activity) as well as on the armless tRNA. The triple variant *Rcu*CCA K74N/K89Δ/V90E shows the same activity as *Rcu*CCA K74N. (**E**) The introduction of the corresponding amino acids of *Rcu*CCA into *Hsa*CCA enables this enzyme to add a complete CCA-end on mt-tRNA^Ile^, in contrast to the wildtype situation. These results indicate that especially position K74 of the β-turn, but to a certain extent also K89/V90, contribute to the substrate adaptation of *Rcu*CCA.

While the β-turn itself (GEKH) is identical in *Rcu*CCA and *Hsa*CCA, the flanking sequences in the *Rcu*CCA enzyme differ in 13 positions from the counterpart, and some of them likely contribute to the extended substrate specificity of this enzyme. In these regions, the *Romanomermis* enzyme carries several lysine residues that are not present in *Hsa*CCA (Figure 5). K74 is also found in the corresponding enzymes of *C. elegans* and *A. suum*, while the human enzyme carries an asparagine at this position (Figure 1). As the basic lysine might enhance the enzymes’ binding to negatively charged tRNA substrates, it represents a top candidate for such a substrate adaptation. At positions 89 and 90, *Rcu*CCA carries a further lysine residue followed by a valine position that might also be involved in tRNA primer binding, although these residues are not present in *Cel*CCA and *Asu*CCA. Hence, we replaced these positions in *Rcu*CCA by the corresponding residues of the human enzyme, resulting in *Rcu*CCA K74N, K89Δ/V90E, and the combination thereof (K74N/K89Δ/V90E). The CCA-adding activity of the recombinantly expressed enzyme variants was determined on yeast tRNA^Phe^ and adjusted to arbitrary units (1 U corresponding to 0.3 ng protein; Figure 5D,E). On this conventional tRNA, all enzymes showed a comparable activity, with a slight reduction in activity of *Rcu*CCA K74N (Figure 5D, upper panel). On mt-tRNA^Ile^, however, the variants carrying the K74N replacement were considerably affected in incorporating the terminal A residue. *Rcu*CCA K89Δ/V90E, in contrast, added a full CCA-end on this tRNA, although it seems to be somewhat less efficient than the wt enzyme. The reciprocal amino acid exchanges and insertions introduced in the human enzyme support these results (Figure 5E). *Hsa*CCA N74K (for reasons of clarity, numbering is according to *Rcu*CCA positions) shows an efficient addition of a complete CCA-end on mt-tRNA^Ile^. Intriguingly, also *Hsa*CCA 89Kins/E90V and the combination N74K/K89ins/E90V exhibit improved CCA-addition on this armless substrate. As a conclusion, K74, and to a certain extent also K89/V90, represent important positions to accept armless tRNA substrates and probably contribute to a stable interaction with the tRNA and the correct positioning of its 3′-end as a primer.

## 3. Discussion

### 3.1. A Specific Adaptation within the Catalytic Core Enables CCA-Addition to Minimalized tRNA Substrates

As the tRNA genes of most organisms do not encode the 3′-terminal CCA-triplet, this essential feature has to be added post-transcriptionally by the CCA-adding enzyme. In eukaryotes, a single enzyme is responsible for this maturation step in both cytosolic as well as organellar tRNA pools [50,51,71]. A second function of CCA-adding enzyme is to monitor the intactness of its tRNA substrate, so that only undamaged molecules are accepted for CCA-incorporation [72,73,74,75,76]. The CCA-adding enzyme does not recognize a specific sequence or base pair in its substrates, but relies on common elements of the overall tRNA cloverleaf structure, as it is also observed for RNase P, tRNase Z, and some tRNA modifying enzymes [77,78,79,80,81]. Metazoan mitochondria, however, encode for tRNAs with deviations from the cloverleaf, where D- or T-arms are reduced or lacking [38,41,48]. As these tRNAs still carry a conventional acceptor stem, they are correctly processed by RNase P, since this enzyme predominantly recognizes this structural feature [78,79]. The CCA-adding enzyme, in contrast, specifically interacts with the tRNA elbow region, and especially with the T-loop region [32,69,82,83,84]. Hence, a bacterial enzyme like the *E. coli* version, evolved for conventional tRNAs, is strongly impaired on truncated tRNAs [52]. The most extreme truncations are observed in nematodes like *R. culicivorax*, in acari and in arachnids, where hairpin-like tRNAs are found that require a specific co-evolution of the corresponding enzymes. On such substrates, the *E. coli* enzyme is completely inactive (Figure 2B). However, the human enzyme co-evolved to accept the D-armless tRNA^Ser^_AGY_ found in human mitochondria (Figure S1) [41]. Hence, this enzyme accepts the armless tRNAs to a certain extent and adds two C-residues, but not the terminal A, indicating that A incorporation requires a specific adaptation to such extreme substrates (Figure 2B, Figure 3, Table 1). In contrast, the *R. culicivorax* enzyme is adapted to these tRNA structures and efficiently adds the complete CCA-triplet. For *Hsa*CCA and *Rcu*CCA, the kinetic parameters for CC-incorporation on standard as well as armless tRNAs are quite similar and correspond to published values [37]. However, when it comes to A-addition, only *Rcu*CCA is adapted to the hairpin-like tRNAs (Figure 2B, Figure 3), although this reaction step is less efficient than C-addition (Table 1).

In the transition from C- towards A-addition, CCA-adding enzymes undergo domain rearrangements to accommodate the growing tRNA 3′-end in the catalytic core and to switch the specificity of the amino acid template in the nucleotide binding pocket from CTP towards ATP recognition [21,22,23,85]. During this structural rearrangement, the tRNA substrate has to remain bound to the enzyme, and this is usually accomplished by specific interactions of the tRNA 3′-end in the catalytic core and of the elbow region (T- and D-loop) with the C-terminal region of the CCA-adding enzyme [22,24]. For CCA-adding enzymes adapted to conventional tRNAs or tRNAs lacking only one arm, this interaction is not very tight, as no K_d_ values could be determined [23,33,69] — yet it is sufficient for a complete synthesis of the CCA-end. However, for armless tRNAs, these interactions seem to be insufficient, and the observed high substrate affinity of *Rcu*CCA (in contrast to the human enzyme; Figure 4, Figure S2) corroborates this hypothesis. Obviously, the *Romanomermis* enzyme is able to bind its substrate very tightly, and since this is also the case for armless tRNAs, this interaction cannot involve the conventional contacts between C-terminus of the enzyme and T-loop of the tRNA but must be located elsewhere, although the C-terminus contributes to the terminal A-addition to a certain extent, as shown by the *Rcu*/*Hsa*CCA chimera A (Figure 4).

In the detailed analysis of enzyme chimeras between *Rcu*CCA and *Hsa*CCA, an adaptation of both enzymes to different tRNA substrates is obvious. In *Hsa*CCA and chimeras B, C, D, and E, the tRNA-binding C-terminus helps to confer an efficient CCA-addition on the conventional tRNA^Phe^ (Figure 3B). As *Hsa*CCA shows a rather weak interaction with tRNA substrates (Figure 4, Figure S2), this C-terminal part obviously contributes to an efficient CCA-synthesis by enabling the final product release [85]. In contrast, in *Rcu*CCA, an adaptation to armless tRNA substrates in the N-terminus is obvious. Here, two elements of the catalytic core are described to play an important role in the specificity switch from C- to A-addition. The flexible loop acts as a lever that adjusts the templating amino acids for correct ATP binding [22,25,26,66], and motif C represents a springy hinge that supports the domain rearrangements involved in this reaction step [22,23]. Accordingly, one could expect that both elements are adapted to the CCA-addition on armless tRNAs. Yet, chimera D (human enzyme with flexible loop and motif C of *Rcu*CCA) does not show any A-addition on the armless tRNA, while it is fully active on a standard substrate (Figure 3), excluding a specific adaptation of these two elements.

A detailed inspection of the residual N-terminal *Rcu*CCA components in the chimeras revealed that constructs carrying a β-turn element located between strands 3 and 4 of the β-sheet in the catalytic core are able to add the terminal A, indicating that this element represents a major adaptation to armless tRNAs (Figure 3B, chimeras B, C, and E)

### 3.2. The ß-Turn Element Impacts the Substrate Affinity of R. culicivorax CCA-Adding Enzyme

In chimera E, the inserted β-turn element consists of 30 amino acids located between motif A and a conserved stretch upstream of the flexible loop (Figure 1). Toh et al., showed that in CCA-adding enzymes for conventional tRNAs, this region plays an important role in binding the growing CC-end of the tRNA and adjusting it in the catalytic core for the nucleophilic attack initiating A-addition [22]. Similar β-turn elements are found for the archaeal class I CCA-adding enzymes [29,30,86], poly(A) polymerases as well as for DNA- and RNA-polymerases, where they are described to position the 3′-hydroxyl of the primer 3′-end in close vicinity of the catalytically important metal ions located in the active site [68]. In *Rcu*CCA, however, the β-turn element has an additional function, as it dramatically contributes to tRNA binding. Here, especially the lysine residue at position 74 (K74) seems to be involved, as its replacement by asparagine, the corresponding position in *Hsa*CCA, affects A-addition on an armless tRNA (Figure 5D). The introduction of K74 into *Hsa*CCA supports the importance of this lysine residue, as it enables a strongly improved CCA-addition on mt-tRNA^Ile^ by this enzyme. Intriguingly, also the *Hsa*CCA K89ins/E90V variant shows an enhanced CCA-addition on mt-tRNA^Ile^. This could be a general effect of the positively charged lysine side chain that keeps the tRNA in close proximity during polymerization, enabling a more efficient nucleotide transfer. In *Rcu*CCA, these positions seem to be less important, as their replacement by the human residues have only very subtle effects on CCA-addition. It is conceivable that other positions not directly involved in tRNA binding might structurally support the substrate adaptation of *Rcu*CCA by folding the loop region into a conformation that optimally positions K74 (and other interacting residues) for primer binding during polymerization. Corresponding crystal structures of *Rcu*CCA in complex with an armless tRNA are needed to clarify this point.

The presented results on *Rcu*CCA, *Hsa*CCA, and the corresponding chimeras support the following hypothesis. As described above, the binding of the tRNA’s T-loop to its C-terminus represents one of the major substrate interactions of the CCA-adding enzyme, ensuring that the tRNA primer remains correctly located for A-addition during the domain rearrangements inducing the specificity switch [34,35]. As a consequence, product release seems to be a limiting factor in the reaction [85]. As this interaction is not possible with armless tRNAs, the human enzyme loses contact after adding the C residues, while a conventional tRNA remains correctly bound in the T-loop/C-terminus interaction and gets elongated by a complete CCA-terminus. In *R. culicivorax*, the CCA-adding enzyme had to adapt to armless tRNAs and evolved a different mode of tRNA interaction, as a binding of T-loop and C-terminus is not possible anymore. Here, the function of the β-turn region evolved from simple primer positioning for A-incorporation into an enhanced high-affinity substrate binding compensating for the loss of the conventional C-terminal tRNA interaction. As this strong interaction is visible at all stages of CCA-addition on canonical as well as armless tRNAs (Figure S2A), the function of the β-turn region in *Rcu*CCA is not limited to bind a tRNA-CC intermediate but generally contributes to an efficient substrate binding during the complete CCA-incorporation reaction. This ensures that the enzyme’s interaction with armless tRNAs is sufficiently strong to survive the structural rearrangements during polymerization. A drawback of this tight binding of *Rcu*CCA might be a reduced product release, and the slightly less efficient CCA-addition on the conventional tRNA^Phe^ could be an indication of this (Table 1).

### 3.3. An Orthogonal Translation System?

Swapping domains between proteins is a useful method to generate proteins with new functions—an approach that is frequently used by nature as well as researchers [87,88,89]. The fact that the replacement of a small β-turn element converts the human tRNA nucleotidyltransferase into an enzyme that accepts armless tRNAs as substrates raises the question whether it is possible to generate an orthogonal system for the incorporation of non-natural amino acids in a host cell system. Representing a fascinating idea, this is highly unlikely, as the armless tRNAs are so different to their conventional counterparts. It is known that many mt-tRNAs are not recognized and charged by cytosolic aminoacyl-tRNA synthetases but require specifically adapted mitochondrial enzymes [90]. Similarly, mitochondrial ribosomes have undergone a specific co-evolution to compensate for the unusual structural features of mt-tRNAs [91]. While metazoan mt-rRNAs are usually shorter than their cytosolic counterparts, the mitochondrial ribosomal proteins are generally enlarged [92,93,94,95]. Again, nematodes represent the extreme case, having ribosomes strongly enriched in protein content but with reduced rRNA components [96,97,98]. Due to this intricate co-evolution of the mitochondrial protein synthesis components, it is probably not feasible to use armless tRNAs as an orthogonal tool in synthetic biology.

Taken together, the CCA-adding enzyme of *R. culicivorax* shows a remarkable adaptation to hairpin-like tRNA where the loss of substrate interactions with the C-terminus is compensated by enhanced tRNA binding of a different enzyme region. The evolutionary plasticity of enzymes is described for the composition of active site residues, where amino acids with identical catalytic roles are located at different positions in the primary sequence [99]. The catalytic core, however, remains unchanged and structurally almost identical. In contrast, the β-turn element of *Rcu*CCA is not a mimicry of the T-loop/C-terminus interaction but recognizes a very different region of the tRNA (the 3′-end) and is still part of the catalytic core. Hence, its specific adaptation to the miniaturized tRNAs add a new layer of evolutionary plasticity. The structural resolution of this enzyme in complex with its tRNA substrate is expected to shed more light into this unusual and fascinating substrate adaptation.

## 4. Materials and Methods

### 4.1. Construction of Recombinant Enzymes

Open reading frames of CCA-adding enzymes from *Escherichia coli* and *Homo sapiens* were cloned into pET30 Ek/LIC plasmid with an N-terminal His_6_-Tag. The mt target signals were not included, and in both enzymes, the cloned coding regions started at the following conserved methionine residue as described [37,50,56,57]. For the CCA-adding enzyme of *Romanomermis culicivorax*, the coding sequence was identified in the *R. culicivorax* genome assembly available at https://parasite.wormbase.org/Romanomermis_culicivorax_prjeb1358/Info/Index/, codon-optimized for expression in *E. coli* and synthesized in pET28a by GenScript^®®^ (Piscataway, NJ, USA). All alignments were done using Jalview 2 [100]. Point mutations were introduced as described [23].

### 4.2. Cloning of Chimeric Enzymes

Chimeric enzymes of CCA-adding enzymes from *Homo sapiens* and *Romanomermis culicivorax* were generated via site-directed mutagenesis in pET30-Ek/LIC or pET28a plasmids, respectively. All chimeras were cloned with an N-terminal His_6_-tag. The fusion positions of all chimeras are shown in Table S1 and Figure S3.

### 4.3. Expression and Purification of Recombinant Enzymes

*E. coli* BL21 (DE3) cca::cam lacking the endogenous CCA-adding enzyme were transformed with plasmids encoding the CCA-adding enzymes from *H. sapiens* (*Hsa*CCA), *R. culicivorax* (*Rcu*CCA), or chimeric enzymes. For CCA-adding enzyme from *E. coli* (*Eco*CCA), *E. coli* BL21 (DE3) was used. Cells were grown in 400 ml LB or TB with 50 µg/ml kanamycin and 35 µg/ml chloramphenicol (only for cca::cam strains) at 30 °C. Expression was induced at OD_600_ = 1.5 by adding 400 ml ice-cold LB or TB containing both antibiotics and IPTG to a final concentration of 1 mM. Cultures were incubated over night at 16 °C and then harvested at 6340 g for 15 min.

Pellets were resuspended in 8 ml ice-cold lysis-buffer (25 mM Tris/HCl pH 7.6, 500 mM NaCl, 1 mM DTT for *Eco*CCA and *Hsa*CCA, 100 mM phosphate buffer pH 7.0, 500 mM NaCl, 10% glycerol, 0.2% NP-40, 1 mM DTT for *Rcu*CCA) and disrupted with 5 g Zirconia beads and Fastprep-24 homogenizer (6 m/s, 30 s). Cell lysates were centrifuged at 30,600 g, 30 min, 4 °C, sterile filtrated and loaded onto a HisTrap FF 1 ml or 5 ml column (GE Healthcare). Column wash was performed with 4–10 column volumes of binding buffer (25 mM Tris/HCl pH 7.6, 500 mM NaCl for *Eco*CCA and *Hsa*CCA, 100 mM phosphate buffer pH 7.0, 500 mM NaCl, 10% glycerol for *Rcu*CCA) with 50 mM imidazole. His-tagged proteins were eluted with 3–8 column volumes of elution buffer (binding buffer with 500 mM imidazole). If necessary, protein containing fractions were further purified by size exclusion chromatography on a HiLoad 16/60 Superdex 75 pg column in binding-buffer containing 200 mM NaCl. Protein-containing fractions were combined and concentrated on Vivaspin 6 columns (15–30 kDa MWCO, GE Healthcare). Proteins were stored in 40% glycerol (*v*/*v*) at −80 °C. Protein concentration was determined according to Bradford [101].

### 4.4. tRNA Preparation

Armless mitochondrial tRNAs for isoleucine and arginine from *R. culicivorax* [48] and canonical cytosolic tRNA^Phe^ from *Saccharomyces cerevisiae* were generated as in vitro transcripts lacking the CCA-end in the presence of α^32^P-ATP (3000 Ci/mmol). Homogeneous 5′- and 3′-ends of the transcripts were generated as described [102]. For kinetic analyses, tRNAs were transcribed without α^32^P-ATP.

### 4.5. Electrophoretic Mobility Shift Assay (EMSA)

0.5 pmol α^32^P-ATP-labeled tRNA substrates were heated for 2 min at 90 °C, cooled to room temperature and incubated with 0 to 4 µM of enzyme in HEPES/KOH (pH 7.6), 30 mM KCl and 6 mM MgCl_2_ at 20 °C for 10 min. After addition of glycerol to a final concentration of 18.5%, tRNAs were separated by 5% native polyacrylamide gel electrophoresis. For visualization of enzyme-bound and free substrates, a Typhoon 9410 scanner (Cytiva, Freiburg, Germany) was used. Dissociation constants were determined in three independent experiments by nonlinear regression using GraphPad Prism 7.

### 4.6. Activity Test and Determination of Arbitrary Units

Initial activity tests for CCA-addition were performed in 30 mM HEPES/KOH pH 7.6, 30 mM KCl, 6 mM MgCl_2_, 2 mM DTT, 0.5 mM NTPs, 5 pmol tRNA, and 20 ng of enzyme in a reaction volume of 20 µl at 20 °C.

For comparative analysis of CCA-addition on armless tRNAs (Figure 3), activity of all enzyme preparations was normalized using canonical tRNA^Phe^ as a substrate. CCA-addition was performed in in the same buffer as described above. For calculation of arbitrary units, 5 pmol of tRNA were incubated with increasing amounts of enzymes for 30 min at 20 °C. Reactions were ethanol-precipitated and analyzed on 10% or 12.5% polyacrylamide gels by autoradiography. Enzyme amounts leading to 50% substrate-turnover were defined as 1 arbitrary unit. Nucleotide incorporation assays were performed as mentioned above. 5 pmol of tRNA were incubated with 1, 5, 10, 25, and 50 arbitrary units of each enzyme preparation for 30 min at 20 °C and analyzed as described [25,58].

### 4.7. Kinetic Analysis

Steady-state Michaelis–Menten kinetics were performed as described [103]. Each reaction contained 1 mM NTPs and 3 µCi of α^32^P-CTP or α^32^P-ATP (3000 Ci/mmol) and 30–35 ng *Hsa*CCA or 30–75 ng *Rcu*CCA. Non-labeled tRNA transcripts without CCA-end were titrated from 1-10 µM and incubated for 15–20 min at 20 °C. Determination of incorporated radioactivity was performed as described [25,66] and kinetic parameters of three independent experiments were calculated by non-linear regression Michaelis–Menten kinetic (GraphPad Prism). As the tRNA transcripts are not soluble at excessive saturating conditions, the calculated kinetic parameters represent apparent values, as frequently used for this type of enzymes [24,25,30,58,65].

### 4.8. Enzyme Modeling

A homology model of *Rcu*CCA enzyme was built using Modeller [104] based on a sequence alignment with the human enzyme and on the human structure (PDB id: 4x4w) [70]. A model of the human enzyme was also built to include all loops that are not visible in the crystal structure. Models were superimposed to the structure of A-adding enzyme:tRNA complex for (PDB id: 4wc2) [34] in PyMOL (v2.4.0, Schrödinger) to position a tRNA in the active site and visualize loops in the vicinity of the tRNA 3′-tail.

## Figures and Tables

**Table 1:** Kinetic parameters of *Hsa*CCA and *Rcu*CCA for CC- and A-addition. As indicated by the relative activity of *Rcu*CCA compared to *Hsa*CCA (change), CC-addition on standard and armless tRNA substrates is similar. In terminal A-addition on the armless tRNAs, *Rcu*CCA shows a 3.3 to 10-fold increase, a clear indication of its adaptation to these substrates. *Hsa*CCA is strongly affected in this reaction, and the obtained low values likely represent an overestimation, as they are close to the detection limit. The actual values are probably much lower. For each analysis, three independent experiments were performed.

Substrate	*Hsa*CCA			*Rcu*CCA			Change
	k_cat_ [s^−1^]	K_M_ [µM]	k_cat_/K_M_	k_cat_ [s^−1^]	K_M_ [µM]	k_cat_/K_M_	(*Rcu*CCA)
**tRNA** ^**Phe**^							
CCA*	0.091 ± 0.012	4.28 ± 1.22	0.02	0.041 ± 0.007	4.66 ± 1.67	0.01	0.5↓
C*C*	0.214 ± 0.034	4.12 ± 1.51	0.05	0.166 ± 0.025	3.01 ± 1.18	0.06	1.2↑
**mt-tRNA** ^**Ile**^							
CCA*	0.006 ± 0.001	2.00 ± 0.95	0.003	0.052 ± 0.011	7.84 ± 2.06	0.01	3.3↑
C*C*	0.165 ± 0.016	5.58 ± 1.08	0.03	0.224 ± 0.042	5.43 ± 2.08	0.04	1.3↑
**mt-tRNA** ^**Arg**^							
CCA*	0.003 ± 0.000	4.77 ± 1.61	0.001	0.012 ± 0.001	1.30 ± 0.56	0.01	10↑
C*C*	0.041 ± 0.004	1.30 ± 0.44	0.03	0.081 ± 0.011	1.69 ± 0.76	0.05	1.7↑

## Supplementary Materials

The following are available online at https://www.mdpi.com/1422-0067/21/23/9047/s1.
