# Supplementary material for: Adaptation of the Romanomermis culicivorax CCA-Adding Enzyme to Miniaturized Armless tRNA Substrates
Source: Int J Mol Sci. 2020 Nov 28;21(23):9047. doi: 10.3390/ijms21239047 (PMC7730189; doi:10.3390/ijms21239047)
Supplement: Supplementary file 1 [file ijms-21-09047-s001.pdf]

Supplementary Material

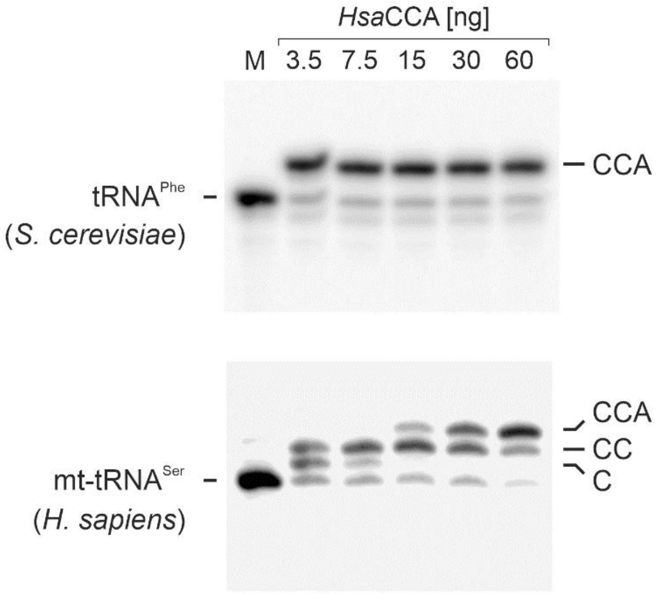

Figure S1. Comparative activity test of *Hsa*CCA on yeast tRNA^{Phe} and human mt tRNA^{Ser}(AGY). To investigate whether the recombinant *Hsa*CCA preparation exhibits its genuine activity on a structurally deviating human tRNA, 5 pmol of the canonical yeast tRNA^{Phe} and the human D-arm lacking mt-tRNA^{Ser}(AGY) were incubated with increasing amounts of *Hsa*CCA as described in Materials and Methods. Both the canonical as well as the D-arm lacking tRNA are accepted for addition of a complete CCA-end, although the human enzyme is less efficient on the mitochondrial tRNA. This experiment clearly demonstrates that the recombinant *Hsa*CCA represents a bona fide enzyme activity that not only tolerates canonical tRNA structures as substrates, but is also adapted to tRNAs of human mitochondria that show some structural deviations. Yet, this adaptation is not sufficient to incorporate a complete CCA-end on armless hairpin-like tRNAs of *R. culicivorax* mitochondria.

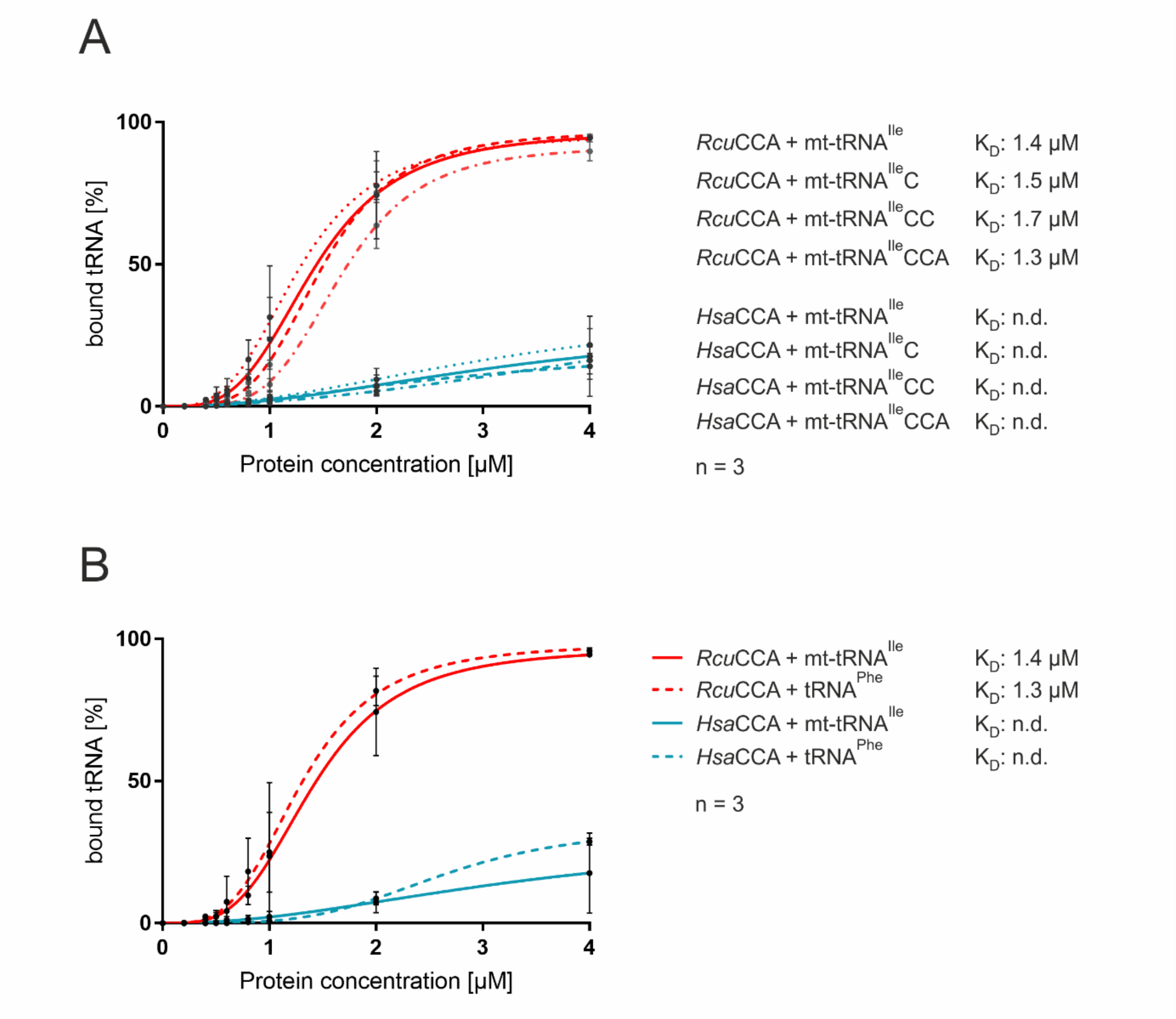

Figure S2. tRNA binding affinities of *Rcu*CCA and *Hsa*CCA. Quantitative analysis of enzyme binding to the armless mt-$tRNA^{Ile}$ and the canonical yeast $tRNA^{Phe}$ was determined by electrophoretic mobility shift. **A.** Binding behavior is not affected by the tRNA 3′-end. All four variants of mt-$tRNA^{Ile}$ are recognized with similar affinities by *Rcu*CCA (red). In contrast, *Hsa*CCA interaction with these tRNAs is too weak to determine binding parameters (cyan). **B.** *Rcu*CCA recognizes armless and canonical tRNAs with similar affinity (red), while both transcripts are only weakly bound by the human enzyme HsaCCA (cyan).

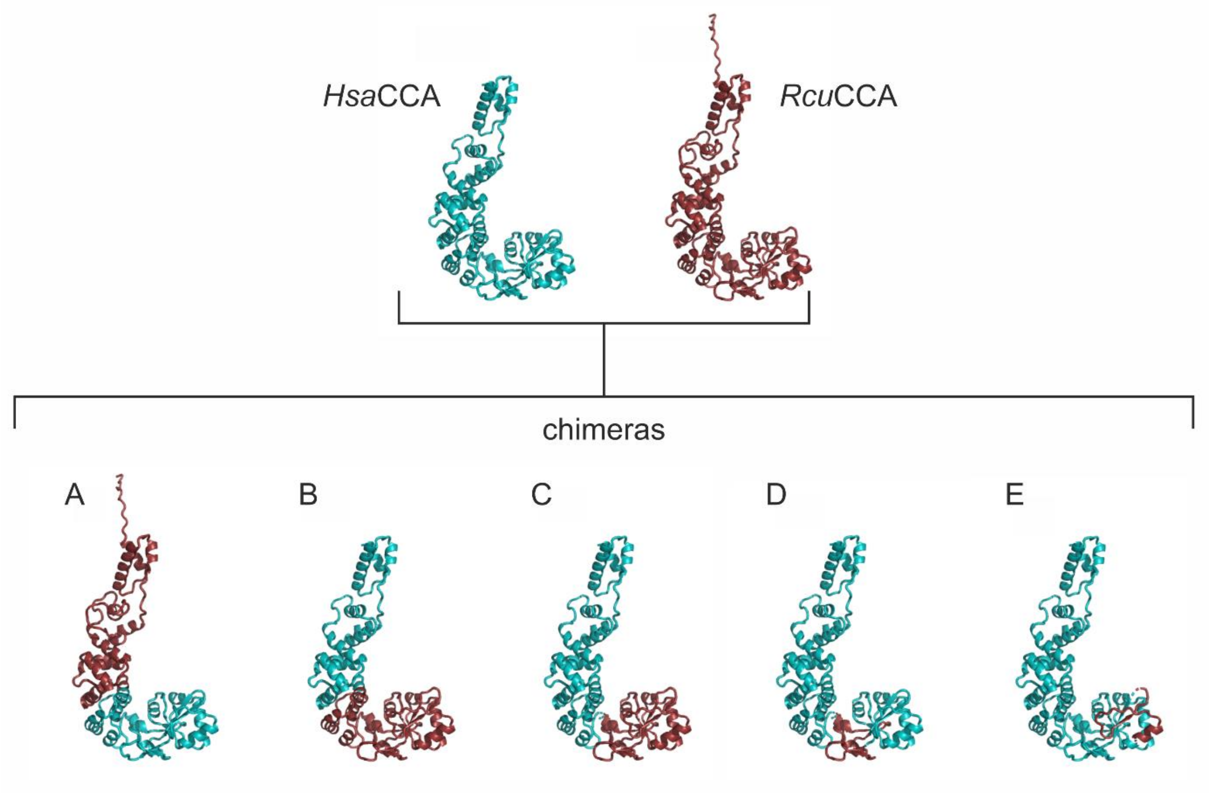

Figure S3. Chimera models. Structural models of *Hsa*CCA (blue) and *Rcu*CCA (red) and chimeras A-E combining regions of native enzymes as described in Figure 3B Table S1.

Table S1. Fusion positions of the investigated chimeras between the human (cyan) and the Romanomermis enzyme (red). Numbering is according to the alignment shown in Figure 1.

**enzyme**	**fusion positions**	**characteristic**
*Hsa*CCA	1-405	*wt*
*Rcu*CCA	1-427	*wt*
chimera A	1-212 / 214-427	*Hsa* N-terminus
chimera B	1-213 / 213-405	*Rcu* N-terminus
chimera C	1-164 / 164-405	*Rcu* N-terminus ending at motif C
chimera D	1-99 / 101-164 / 164-405	*Rcu* flexible loop and motif C region
chimera E	1-60 / 61-91 / 91-405	*Rcu* β-turn element
